# Deep Learning in Phosphoproteomics: Methods and Application in Cancer Drug Discovery

**DOI:** 10.3390/proteomes11020016

**Published:** 2023-05-02

**Authors:** Neha Varshney, Abhinava K. Mishra

**Affiliations:** 1 Division of Biological Sciences, Department of Cellular and Molecular Medicine, University of California, San Diego, CA 93093, USA; 2 Ludwig Institute for Cancer Research, La Jolla, CA 92093, USA; 3 Molecular, Cellular and Developmental Biology Department, University of California, Santa Barbara, CA 93106, USA

**Keywords:** phosphoproteomics, machine learning, deep learning, cancer, post-translational modification, personalized medicine

## Abstract

Protein phosphorylation is a key post-translational modification (PTM) that is a central regulatory mechanism of many cellular signaling pathways. Several protein kinases and phosphatases precisely control this biochemical process. Defects in the functions of these proteins have been implicated in many diseases, including cancer. Mass spectrometry (MS)-based analysis of biological samples provides in-depth coverage of phosphoproteome. A large amount of MS data available in public repositories has unveiled big data in the field of phosphoproteomics. To address the challenges associated with handling large data and expanding confidence in phosphorylation site prediction, the development of many computational algorithms and machine learning-based approaches have gained momentum in recent years. Together, the emergence of experimental methods with high resolution and sensitivity and data mining algorithms has provided robust analytical platforms for quantitative proteomics. In this review, we compile a comprehensive collection of bioinformatic resources used for the prediction of phosphorylation sites, and their potential therapeutic applications in the context of cancer.

## 1. Introduction

Protein phosphorylation is the most widespread post-translational modification (PTM) in eukaryotes and plays a cardinal role in regulating protein functions, such as modulating their intracellular dynamics, stability, subcellular localization, and interaction with other proteins [1,2]. Protein phosphorylation is reversibly controlled by protein kinases (PK) and protein phosphatases (PP) [3]. Protein phosphorylation regulates many cellular processes, including cellular metabolism, cell migration, cell division, proliferation and differentiation, apoptosis, etc. [4,5,6,7,8,9,10,11]. Dysregulated phosphorylation has been identified as a hallmark of many diseases, including numerous cancers, Alzheimer’s disease, and diabetes [12,13,14]. Therefore, understanding protein phosphorylation and its effects on cell signaling is a major endeavor in the post-genomics era.

Recent advances in experimental approaches have immensely helped in the characterization of PTMs. However, the analysis and understanding of PTMs involve several challenges. Efficient and sensitive methods for the detection of PTMs are indispensable. Traditionally, techniques including Edman degradation, mutational analysis, isotopic labelling, or immunochemistry have been used for PTM such as protein phosphorylation discovery [15,16,17]. Recently, mass spectrometry (MS)-based approaches have shown to be useful in protein phosphorylation identification [18]. MS provides a good platform for the experimental determination of protein phosphorylation sites and high in-depth coverage, and it provides opportunities for ML-based approaches to handle large datasets in public repositories. PTM research has made remarkable progress over the years, especially after the emergence of new computational techniques. Combined with experimental methods, the application of bioinformatics tools in PTM analysis enables a more efficient exploration of the phosphorylation network, resulting in the timely analysis of datasets and providing insights for biological research and drug discovery [19]. 

Deep learning (DL) in phosphoproteomics refers to the application of machine learning (ML) algorithms to analyze large amounts of data generated from phosphoproteomic experiments. The aim of ML is to identify patterns, classify proteins, and make predictions about protein phosphorylation. The data analysis in phosphoproteomics involves the identification of phosphopeptides based on MS/MS spectra. This can be performed by database searches. The databases report phosphopeptide sequences along with assigned phosphorylation sites. Next, to determine the confidence of each possible phosphorylation site candidate in an identified peptide sequence, several computational algorithms or ML-based approaches can be used. A global understanding of the protein phosphorylation network using these approaches can aid in our understanding of cellular signaling pathways, disease mechanisms, disease onset prediction, drug development, and therapy response in an efficient yet comprehensive manner.

In this brief review, we survey the mainstream tools available to explore the phosphorylation network. Additionally, we present a comparative analysis of these computational tools in terms of technique used, implementation, performance, functionality and limitations from the perspective of a biologist. Finally, we discuss the applications of these phosphoproteomics-based bioinformatics tools in cancer research in identifying novel drug targets and advancing personalized medicine. Hence, this review aims to bridge the gap and emphasize the complementarity between traditional MS-based methods to study phosphoproteomics and the new cutting-edge deep-learning-based prediction methods.

## 2. Methods for Phosphorylation Site Prediction

The computational approaches provide a promising strategy for identification and understanding of phosphorylation sites. Several computational methods have been developed for phosphorylation site prediction over the years. These can be classified into two main categories: algorithm-based and more advanced ML-based methods. 

### 2.1. Algorithm-Based Computational Approaches

In the past, many studies used algorithm-based computational methods to predict phosphorylation sites in which there are no learning algorithms used to gain information directly from data. They can be further classified into simple consensus pattern-based approaches (SCPs) and sequence similarity-based clustering methods (SSs). For example, in 1988, one of the first computational approaches to predict PTM sites was developed, which used the primary sequence of the protein and SCP approach [20]. Other examples of SCPs are PROSITE [21], ELM [22], and HPRD [23], which depend upon the presence of an exact motif surrounding the phosphorylation site. However, SSs-based methods were later designed to provide a high score to a query peptide that has a high similarity score with known phosphorylation peptides, using the sequence similarity measures such as the BLOSUM62 matrix. PostMod [24] and PSEA [25] are examples of this category. These methods have been shown to be inappropriate for large-scale analyses since the performance of these methods in predicting phosphorylation sites is poorer than more advanced ML-based approaches. 

### 2.2. Machine Learning (ML)-Based Computational Approaches

Over the last decade, the integration of ML into a wide range of computational models has improved prediction accuracy and gained a better understanding of protein function and PTMs [26,27]. With the explosion of DL methods, ML-based approaches for phosphorylation site prediction have become more popular. ML is generally the ability of machines to do actions based on prior knowledge and experience [28]. ML-based methods can learn the underlying rules and signatures in the data by tuning and optimizing related parameters during the model training process, resulting in better performance as compared to SCP-based methods. A few examples of ML-based techniques for phosphorylation site prediction are neural network (NN), hidden Markov models (HMM), Bayesian decision theory (BDT), support vector machines, logistic regression (LR), random forest (RF), K-nearest neighbor (KNN), and conditional random fields (CRFs) [29,30,31]. A few examples of phosphorylation site prediction tools based on these techniques are NetPhos, KinasPhos, DISPHOS, and Ptpset. Most of the databases and phosphorylation-site prediction tools that use different algorithms and ML-based approaches are listed in Table 1 and Table 2, respectively. The development of these models have set the benchmark for ML- and DL-based approaches for various PTM predictions. 

## 3. Framework of ML-Based Approaches for Phosphorylation Site Prediction

**Figure 1:**
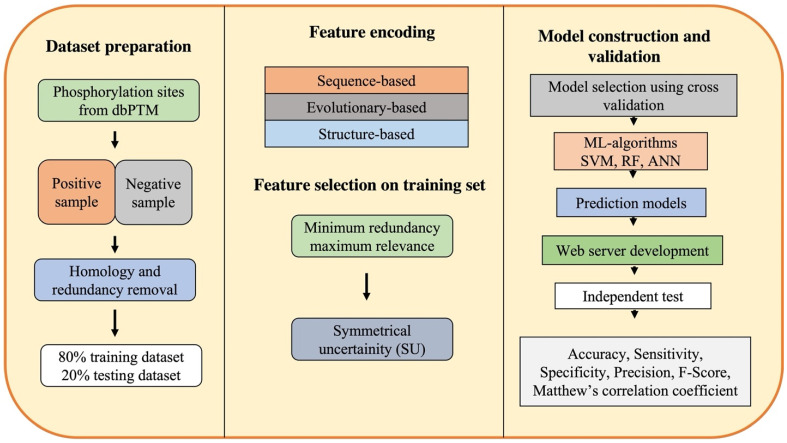
Workflow of machine learning-based computational approaches for phosphorylation site prediction (SVM: support vector machine, RF: random forest, ANN: artificial neural network).

Generally, ML-based computational approaches for phosphorylation site prediction are developed using the following five steps: (1) dataset preparation; (2) selection of encoding methods; (3) building prediction models; and (4) performance evaluation and development of a web-server (Figure 1). 

### 3.1. Dataset Preparation

The first step for phosphorylation site prediction is dataset preparation that includes the extraction of experimentally validated phosphorylation sites from the publicly available databases, dbPTMs, and the literature [86]. A few of the protein phosphorylation databases are enlisted in Table 1. An extracted dataset must include both positive and negative datasets. The fragments or peptides that have the phosphorylated residues (S, T, Y) compiled from the aforementioned dbPTMs are considered a positive dataset. The S, T, Y amino acids in the experimental peptides with no phospho-groups on them are considered as negative datasets. Almost all studies use databases to gather positive samples, yet, selecting the negative dataset is the most challenging step. While a particular residue that can be phosphorylated can be validated experimentally, a particular residue that is not phosphorylated under any conditions is difficult to prove experimentally. Therefore, databases contain thousands of known phosphorylation sites but do not contain phosphorylation sites that are known to be unphosphorylated. A few criteria to apply while extracting a negative dataset include the selection of a site that should not have been reported as a phosphorylation site in the positive dataset, the thresholding of a solvent accessible area of the protein, etc. Following the construction of these datasets, the next step is the removal of homologous and redundant sequences. The Cluster Database at high identity with tolerance (CD-hit) is a popular program to detect and filter similar sequences [87]. The threshold of identity between sequences is considered to range from 30% to 90%. These prepared datasets are used as benchmark data, which are eventually divided into 80% training data for learning and 20% testing set for model validation. The training data are used for feature selection and ML model generation, which also includes a 5-fold internal cross-validation of the trained classifiers’ performance. The testing dataset is used for further assessment and validation of ML models using various statistical measures. 

### 3.2. Feature Encoding and Selection

For feature encoding, all the proteins are partitioned into polypeptides in such a way that the target residue is placed at the center of the peptide. Each polypeptide sequence (both positive and negative datasets) is encoded as a numerical feature vector according to the appropriate biological descriptors, such as amino acid composition [88], similarity score to the known motifs [89], and evolutionary and structural properties [90]. Occasionally, to enhance the prediction performance, all features are pooled, thus resulting in a combination of features to generate learning models. Feature selection methods are then used to choose the most relevant features while minimizing the redundancy in the data and further improving the model performance by reducing its computational time. The feature selection is performed at two levels: minimum redundancy maximum relevance (mRMR) approach followed by symmetrical uncertainty (SU) selection method. mRMR is a widely used feature selection method approach that ranks the features while taking into consideration their importance to the classification variable along with the redundancy among the features themselves [91]. The SU attribute evaluation method weighs the merit of an attribute by determining its uncertainty with reference to other sets of attributes [92].

### 3.3. Model Construction and Validation

Once the features have been extracted, data are used to train a model/classifier for PTM site prediction. At this point, different classifiers are trained, and based on the performance of each classifier, a suitable classifier is selected. One of the most popular ML-based methods used for predicting sites is SVM. SVMs are a set of points in the n-dimensional space of data that define the boundaries of categories. It is a maximum margin classifier in which data are separated by a hyperplane, provided that they have the highest margin over the data. RF is one of the other well-known ML-based algorithms used for phospho-sites. RF is a supervised learning algorithm; as the name suggests, it builds forests randomly whereby forests are groups of decision trees. Once several decision trees are made, they are merged to make more stable and accurate stable predictions. The classifier is trained on a subset of assembled dataset (training dataset) after parameter optimization and, finally, the predictor is ready to be assessed for performance and compared with other methods. The prediction performance of the model is assessed by its accuracy (proportion of correct positive and negative predictions), sensitivity or true-positive rate, F-score, and Mathew’s correlation coefficient (MCC). An independent test set is carried out to evaluate the performance of the classifier and further verify its practicality.

## 4. Use of Machine Learning-Based Approaches for Phosphoproteome Prediction in Cancers

**Figure 2:**
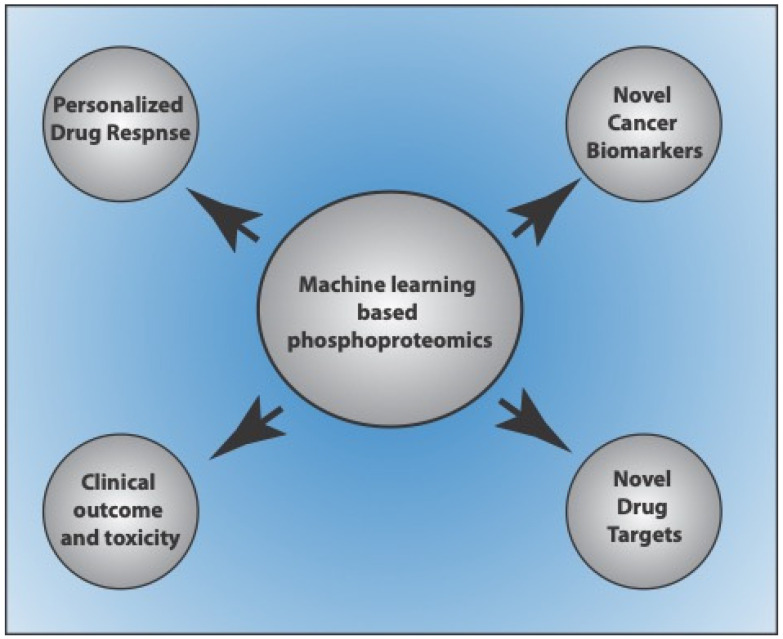
Application of machine learning-based phosphoproteome prediction studies in cancer.

Quantitative phosphoproteomics-based approaches are powerful tools to investigate the signaling pathways and cross-talk networks in cancer cells, assess disease prognosis, and develop personalized treatments [8,9,93,94,95]. Integrating ML and multi-omics data to classify cancer stages or accelerate the prognosis of the disease in the early stages is an active area of investigation. Many in silico approaches for predicting the phosphoproteomic profiles of cancer patients have gained attention in recent years. Sequence-based approaches to predict phosphoproteomes have limited accuracy as phosphoproteomic profiles may vary considerably across cancer patients [96]. Further, MS-based approaches are time-consuming and expensive. Therefore, new computational methods to predict phosphoproteomic profiles across cancer patients are now widely investigated. Several models have been developed and used to predict phosphoproteome in cancer cells, discover biomarkers, patient-specific drug targets, individualized prediction of drug response, and clinical outcomes and toxicity [95,97,98,99,100,101] (Figure 2). 

### 4.1. Machine Learning-Based Approaches for Phosphoproteome-Based Biomarker Prediction

The Cancer Genome Atlas (TCGA), the National Cancer Institute (NCI), and the Clinical Proteomic Tumor Analysis Consortium (CPTAC) are valuable resources that provide a collection of genomic, transcriptomic, proteomic, and phosphoproteomic data for a variety of cancer types. Artificial intelligence (AI) can be used to train these datasets to create algorithms that can predict patient-specific outcomes by predicting biomarkers. For example, using the Boruta algorithm to identify mutant genes involved in the vascular invasion from TCGA, the National Institute of Health, Medical Research, and AMC databases, a gene signature was identified and a recurrence prediction model for recurrence for HCC patients was established [102]. A convolutional NN algorithm was used to analyze proteomics and histology imaging datasets generated by the Clinical Proteomic Tumor Analysis Consortium (CPTAC) from clear cell renal cell carcinoma patients. This study reported a robust correlation between diagnostic markers and predictions generated by the imaging-trained classification model [103]. Joint learning (JL) is a type of ML method to predict proteome from the transcriptome. This method was developed using a training dataset by NCI-CPTAC and TCGA, consisting of proteomic, phosphoproteomic, and transcriptomic data from 77 breast and 105 ovarian cancer samples. In this powerful model, a gene-specific regulatory network was trained, followed by creating a cross-tissue model by JL, the shared regulatory networks and pathways across many cancer tissues. Such a robust model can help facilitate biomarker discoveries for high- and low-risk patients in survival analyses with different clinical outcomes due to the activation of different functional pathways [104]. Further, the proteome complexity across cancer types and within the patient-specific cohort can also be effectively studied using these models, whereas the traditional approaches may have limited scope to address these issues. 

Further, to predict the drug response and design rational combination therapies, a recent study used seven targeted anticancer drugs in 35 non-small cell lung cancer (NSCLC) cell lines and 16 samples of pleural effusions from NSCLC and analyzed dynamic changes in 52 phosphoproteins. They developed an orthogonal ML approach to predict drug response and rational combination therapy. Such studies can supplement the existing methods of using gene mutations to predict biomarkers by utilizing the proteomics data and predict treatment choices and therapy outcomes based on the dynamic proteome complexity [98]. 

### 4.2. Machine Learning-Based Approaches for Phosphoproteome-Based Patient-Specific Drug Targets and Responses

ML is becoming increasingly popular and valuable in enhancing our current understanding of established or new molecular targets in regulating stemness and cancer metastasis. These studies are key to identifying novel phosphoproteome-based drug targets for hard-to-treat cancers. In a recent in-depth global and phosphoproteomic analyses of tumor cells, using protein structure modeling and interface prediction-guided mutagenesis, the interaction between CD44 and CD81 in extracellular vesicles (EVs) secretion was identified [100]. EVs are the drivers of breast cancer stemness and metastasis in triple-negative breast cancer (TNBC). Hence, this study is seminal to identifying new molecular drug targets with the help of ML approaches. Another study analyzed the phosphoproteomes of cholangiocarcinoma cell lines and patient tumors using MS-based phosphoproteomics and computational methods to identify patient-specific drug targets. This study identified the inhibitors of histone deacetylase and PI3K pathway members as high-ranking therapies to use in primary cholangiocarcinoma by the drug ranking using machine learning (DRUML) algorithm [97]. Drug ranking using ML (DRUML) has also been successfully applied to predict the efficacy of anticancer drugs [105].

KSTAR is graph- and statistics-based algorithm that can capture patient-specific kinase activities from phosphoproteomic data. This algorithm was applied to clinical breast cancer phosphoproteomic data. The study reported that the predicted kinase activity profiles could successfully identify misclassified HER2-positive breast cancer patients. In addition, the algorithm can also identify the likelihood of clinically diagnosed HER2-negative patients to respond to HER2-targeted therapy [106]. Thus, in addition to identifying novel drug targets, ML-based studies are also actively contributing to our current understanding of patient-specific drug responses. 

Cellular immunotherapies are a form of personalized medicine that has revolutionized cancer treatment. However, only a subset of patients responds to immunotherapy; hence, there is vast room for improvement. In a recent study, ML-based algorithms were applied to MS-based serum proteomics signatures to predict the response and toxicity of immunotherapy. Datasets from advanced non-small cell lung cancer and malignant melanoma patients were used in this study. Interestingly, the algorithm was able to effectively categorize patients into groups with good and poor treatment outcomes independent of the biomarker signatures [99].

To understand the disease progression and therapy outcome and to identify new drug targets, a holistic understanding of the complex phosphoproteome in cancer is required. This will involve a combination of mass spectrometry-based phosphoproteomics, together with databases and bioinformatics tools to capture the actual, real-time activity of kinases. Such tools could be valuable to establish a phosphoproteomics-based personalized medicine platform for hard-to-treat cancers.

## 5. Conclusions and Future Perspective

The function of a protein is strongly affected by the post-translational chemical modifications that play important functions in a myriad of cellular processes. Therefore, PTM identification is critical for the understanding of molecular functions and diseases. The considerable amount of PTM data generated from the in-depth MS-based experimental approaches could be used to support the development of downstream computational identification methods. DL is a highly effective computational approach to understand large and complex datasets to predict PTMs. In recent years, several DL methods have been developed to predict PTM sites with high efficiency. While these tools have shed light on the quicker, efficient, and less labor-intensive ways on the discovery of phosphorylation site prediction, there are some common weaknesses in assessing these methods, and various factors should be considered in deciding which tool to choose. The most critical factor relevant for the evaluation of prediction tools is the motif size and proper biological context. Another important factor relevant for consideration in PTM predictor construction is the quality of underlying data, including the amount and redundancy of example substrate protein sequences and the level of authenticity. There are several DL algorithms employed; however, each model has its own advantages and disadvantages. In many models, PTM sites are predicted based on sequence information, physical properties, chemical properties, and protein structure properties, but there is still room for approaches that are based on reduced amino acid compositions [107,108,109].

Thousands of phosphorylation sites have been identified for different proteins by MS; however, the kinase responsible for the phosphorylation of that amino acid in a few of the reported datasets is missing. Therefore, there is a need to develop databases which could bridge the gap between the number of experimentally identified phosphorylation sites and the number of phosphorylation sites for which the modifying kinase is known. While PTM identification can be implemented with DL-based methods in a non-invasive, efficient, and low-cost way, there is still a caveat if these algorithms can be directly used for diseases diagnoses. The over-arching problem is the false-positive rate, which is not ideal for its application in healthcare studies where every misdiagnosis can pose a danger to a patient’s health. An ideal model is characterized by high sensitivity and a very low false-positive prediction rate. Therefore, further research is required to evaluate more state-of-the-art frameworks so that these techniques could be applied in clinical practice more effectively. 

A phosphorylation event is dynamic and cell type-specific and cannot be traced in a heterogenous cell population, highlighting the importance of analyzing phosphorylation events at the single-cell level for complex samples, such as tissues and organs. With the advent of single-cell proteomics, the adaptation of phosphoproteomics profiling to single-cells has revolutionized the field in uncovering the heterogeneity in signaling networks, complementing single-cell genomics and transcriptomics [110,111,112]. Therefore, we believe that an integration of computational and biochemical approaches will form the basis for the future development of methods that can reconstruct trans-regulatory networks for heterogeneous cells in single-cell multi-omics data [113]. Another forth-coming area of research in this field is the characterization of cross-talk between different types of PTMs.

Mass spectrometry is one of the key platforms for proteomic analyses that involves either a ‘bottom-up’ or a ‘top-down’ proteomics approach. The traditional ‘bottom-up’ approach employs the digestion of intact proteins into peptides, followed by introduction into the mass spectrometer for fragmentation/detection. Majority of the ML-based methods run smoothly on the bottom-up proteomics data. In the ‘top-down’ approach, the proteins are ionized directly and the intact fragmented proteins rather than digestive peptides are used in the analysis [114]. Many phosphoproteins have been studied using the top-down approach [115,116,117]. However, one of the major challenges in top-down proteomics data analysis is the complexity of the high-resolution top-down mass spectra that involves centroiding, deconvolution, proteoform identification, and quantification [118]. A number of algorithm- and ML-based approaches are now actively being developed to enhance the predictions in the top-down proteomics. These methods will be extremely valuable resources that will aid into our understanding of proteoform complexity and improve the performance of disease diagnosis and drug target discovery. 

Recently, ensembled learning-based feature selection methods were employed to explore the nature of the phosphorylation of SARS-CoV-2 to contribute to SARS-CoV-2 drug discovery [119]. Finally, in the era of personalized medicine, ML-based approaches in phosphoproteome studies will play an instrumental role both in understanding the disease mechanisms and in identifying new therapy targets. ML-based approaches will be valuable in discovering novel biomarkers, advance our current understanding of patient-specific drug targets and drug responses, and facilitate cancer stage classification.

## Figures and Tables

**Table 1:** List of protein phosphorylation databases.

Name	Technique	Organisms	Description/Functionality	Ref	Website
			Kinome		
Kinomer	HMMER 2.3.2	Eukaryotes	Annotated classifications for the protein kinase complements of 43 eukaryotic genomes	[32,33]	http://www.compbio.dundee.ac.uk/kinomer/ (accessed on 24 April 2023)
KinaseNET	-	Human	Comprehensive source on human kinases	-	http://www.kinasenet.ca/ (accessed on 24 April 2023)
			Phosphatome		
PTP	-	-	Integrates sequence and structure with cellular and biological functions on protein tyrosine phosphatases	[34]	http://ptp.cshl.edu/ (accessed on 24 April 2023)
DEPOD	BLAST		Comprehensive and informative database on human kinase-phosphatase substrate	[35]	http://depod.bioss.uni-freiburg.de/ (accessed on 24 April 2023)
			Kinome-Phosphatome		
Phospho.ELM	BLAST	Multiple	Database designed to store in vivo and in vitro phosphorylation	[36]	http://phospho.elm.eu.org/ (accessed on 24 April 2023)
PSP (PhosphositePlus)	-	Human, mouse, and rat	Resource that comprehensively curates information about the structure and regulatory interactions of phosphorylation sites	[37]	https://www.phosphosite.org (accessed on 24 April 2023)
UniProt	-	Multispecies	A central hub for the collection of functional information on proteins	[38]	https://www.uniprot.org (accessed on 24 April 2023)
EPSD (Eukaryotic Phosphorylation site Database)	-	Multiple	A data resource for the collection, curation, integration, and annotation of p-sites in eukaryotic proteins	[39]	http://epsd.biocuckoo.cn (accessed on 24 April 2023)
RegPhos 2.0 (regulatory network in protein phosphorylation)	-	Human, mouse, and rat	A comprehensive tool to view intracellular signaling networks by integrating the information of metabolic pathways and protein–protein interactions	[40]	http://140.138.144.141/~RegPhos/ (accessed on 24 April 2023)
Phospho3D 2.0	-	Multiple	A database for the collection of information on the residues surrounding the p-site in space (3D zones)	[41]	http://www.phospho3d.org/ (accessed on 24 April 2023)
dbPSP	-	Prokaryotes	Collection of p-sites in prokaryotic phosphoproteins	[42]	http://dbpsp.biocuckoo.cn (accessed on 24 April 2023)
LymPHOS	-	Human, mouse	A database for storage, sharing, and visualization of data related with the human T-lymphocyte phosphoproteome	[43]	http://www.lymphos.org (accessed on 24 April 2023)
P3DB	-	Plant species	Displays data in a relational, hierarchical manner that integrates proteins, peptides, phosphosites, and spectra for each phosphorylation event	[44]	http://www.p3db.org/ (accessed on 24 April 2023)
PHOSIDA (Phosphorylation site database)	-	Multiple	Structural and evolutionary investigation and prediction of phosphosites	[45]	http://phosida.de/ (accessed on 24 April 2023)
HPRD (human protein reference database)	BLAST	Human	A database of curated proteomic information including PTMs, kinase/phosphatase motifs, and binding motifs pertaining to human proteins	[46]	http://www.hprd.org (accessed on 24 April 2023)
VPTMdb	SVM, NB, RF	Virus	Predicts viral p-Ser	[47]	http://vptmdb.com:8787/VPTMdb/ (accessed on 24 April 2023)
pTestis	-	Mouse	Testis phosphorylation sites from various studies were analyzed, integrated with the iGPS prediction results, which present the potential kinase–substrate regulatory relationships	[48]	http://ptestis.biocuckoo.org/ (accessed on 24 April 2023)
PhosphoPep	BLAST	Multiple	Database of protein phosphorylation sites for systems level research in model organisms	[49]	http://www.unipep.org/phosphopep/index.php (accessed on 24 April 2023)
PhosphoPOINT	PPI, BLASTP	Human	Annotates interactions among kinases, with their downstream substrates and interacting phosphoproteins	[50]	http://kinase.bioinformatics.tw/ (accessed on 24 April 2023), https://bioregistry.io/registry/phosphopoint.protein (accessed on 24 April 2023)

**Table 2:** A comprehensive list of computational tools used for phosphoproteomic data analysis, including phosphorylation site prediction, predicting kinases, and phosphoproteomic data annotation. Column headings are as follows. Name: Name of the tool; Technique: the machine learning technique used; Organisms: list of organisms the tool is applicable; Description/Functionality: important properties of the tool in terms of its functions; Reference; the paper describing that tool, website: the address of that tool’s web implementation or source of access (if applicable). ANN: artificial neural network; PSSM: position-specific scoring matrices; ZSL: zero-short learning; RNN: recurrent neural network; BLR: bagged logistic regression; LR: logistic regression algorithm; DNN: deep neural network; PPI: protein–protein interaction; XGBoost: extreme gradient boosting; MLS: motif length selection (MLS); LSTM: long short-term memory.

Name	Technique	Organisms	Description/Functionality	Ref	Website
NetPhos 3.1	ANN	Multiple	Generates NN predictions for serine, threonine, and tyrosine phosphorylation sites in eukaryotic proteins. Utilizes sequence composition features, both generic and kinase specific predictions	[30]	https://mybiosoftware.com/tag/netphos (accessed on 24 April 2023)
NetPhosK	ANN	Eukaryotes	Kinase-specific phosphorylation sites prediction	[51]	https://www.hsls.pitt.edu/obrc/index.php?page=URL1117048165 (accessed on 24 April 2023)
Scansite 2.0	PSSM	Human	A tool built on experimental binding and/or substrate information from oriented peptide library screening and phage display experiments, together with detailed biochemical characterization to derive a weight matrix-based scoring algorithm that predicts protein–protein interactions and sites of phosphorylation	[52]	https://scansite4.mit.edu/#home (accessed on 24 April 2023)
PhosphoNet	PSSM	Human	An open source of putative phosphosites predicted after improvisation of kinase substrate prediction algorithm to the primary structure of proteins	[53]	http://www.phosphonet.ca (accessed on 24 April 2023)
Predphospho	SVM	Human	Predicts the changes in phosphorylation sites caused by amino acid variations at intra- and interspecies levels	[54]	http://www.ngri.re.kr/proteo/PredPhospho.htm (accessed on 24 April 2023)
NetworkKIN	ANN, PSSM	Human	Uses probabilistic protein association network (string) to model the context of kinases and substrates, combined with consensus sequence motifs	[55]	https://networkin.info/ (accessed on 24 April 2023)
jEcho	Weight vector	Human	Phosphorylation sites of kinases	[56]	http://www.healthinformaticslab.org/supp/resources.php (accessed on 24 April 2023)
PhoScan	Scoring function	Human	Predicts kinase-specific phosphorylation sites with sequence features by a log-odds ratio approach	[57]	http://bioinfo.au.tsinghua.edu.cn/phoscan/ (accessed on 24 April 2023)
Predphos	SVM	Multiple	Structural-based prediction of phosphorylation sites, hybrid approach, which incorporates bootstrap resampling technique, SVM-based fusion classifiers and majority voting strategy	[58]	No tool link
NetPhosYeast	ANN	Yeast	Prediction of protein phosphorylation sites in yeast	[59]	https://services.healthtech.dtu.dk/service.php?NetPhosYeast-1.0 (accessed on 24 April 2023)
GPS 6.0 (group-based prediction system)	MLS, PSSM, GA	Mammalian	Protein phosphorylation sites and their cognate kinases (addresses false positive rates in prediction)	[60,61]	http://gps.biocuckoo.org/ (accessed on 24 April 2023)
iGPS	GPS with PPI	Human	It is a GPS algorithm with the interaction filter, or in vivo GPS mainly for the prediction of in vivo site-specific kinase-substrate relation (ssKSRs)	[62]	http://igps.biocuckoo.org/links.php (accessed on 24 April 2023)
PPRED	PSSM, SVM	-	Incorporates only evolutionary information of PSSM profile of the proteins in predicting phosphorylation sites	[63]	http://www.cse.univdhaka.edu/~ashis/ppred/index.php (accessed on 24 April 2023)
Phos3D	SVM	Human	Prediction of phosphorylation sites (p-sites) in proteins, originally designed to investigate the advantages of including spatial information in p-site prediction	[64]	https://phos3d.mpimp-golm.mpg.de/cgi-bin/index.py (accessed on 24 April 2023)
DAPPLE 2	BLAST	Human	Homology-based prediction of phosphorylation sites	[65]	http://saphire.usask.ca/saphire/dapple2 (accessed on 24 April 2023)
EMBER	CNN + RNN	Multiple	Embedding-based multilabel prediction of phosphorylation events (EMBER), a DL method that integrates kinase phylogenetic information and motif-dissimilarity information into a multilabel classification model for the prediction of kinase motif phosphorylation events	[66]	https://github.com/gomezlab/EMBER (accessed on 24 April 2023)
KinomeXplorer	NetworKIN algorithm, a novel Bayesian scoring scheme	Human and major eukaryotes	Analyze phosphorylation-dependent protein interaction networks	[67]	http://kinomexplorer.info/ (accessed on 24 April 2023)
PhosTransfer	CNN	Info not available	Hierarchical kinase-specific phosphorylation site (KPS) prediction	[68]	https://github.com/yxu132/PhosTransfer (accessed on 24 April 2023)
MusiteDeep	CNN/CapsNet	Human	Prediction and visualization for multiple PTMs and simultaneously potential PTM cross-talks	[69]	https://www.musite.net (accessed on 24 April 2023)
PROSPECT	CNN	*E. coli*	Predicts histidine phosphorylation sites from sequence information	[70]	https://bio.tools/prospect-web (accessed on 24 April 2023)
DeepKinZero	ZSL	Human	Predicts the kinase acting on a phosphosite for kinases with no known phosphosite information	[71]	https://github.com/Tastanlab/DeepKinZero (accessed on 24 April 2023)
DeepPPSite	LSTM	Mammals and *Arabidopsis thaliana*	Long short-term memory (LSTM) recurrent network for predicting phosphorylation sites	[72]	https://github.com/saeed344/DeepPPSite (accessed on 24 April 2023)
DeepIPs	CNN + LSTM	Human	Identification of phosphorylation sites using deep learning method	[73]	https://github.com/linDing-group/DeepIPs (accessed on 24 April 2023)
Rice_Phospho 1.0	SVM	Rice	Predicts protein phosphorylation sites in rice	[74]	http://bioinformatics.fafu.edu.cn/rice_phospho1.0 (accessed on 24 April 2023)
Yeast KID	-	Yeast	The first literature-curated database for kinases that integrates a series of HTP and LTP, genetic, physical, and biochemical experimental evidence with the goal of establishing known kinase–substrate relationships.	[75]	http://www.moseslab.csb.utoronto.ca/KID/ (accessed on 24 April 2023)
AutoMotif	SVM		The service uses a supervised support vector machine approach to predict various types of phosphorylation sites in proteins	[76]	http://ams2.bioinfo.pl/ (accessed on 24 April 2023)
PhosIDN	Multilayer DNN	Human	An integrated DNN approach for improving protein phosphorylation site prediction by combining sequence and protein–protein interaction information	[77]	https://github.com/ustchangyuanyang/PhosIDN (accessed on 24 April 2023)
DeepPhos	CNN	Human	Uses densely connected CNN for kinase-specific phosphorylation site prediction	[29]	https://github.com/USTC-HIlab/DeepPhos (accessed on 24 April 2023)
Chlamy-EnPhosSite	CNN + LSTM	*Chlamydomonas reinhardtii*	Can predict novel sites of phosphorylation within the entire C. reinhardtii proteome	[78]	https://github.com/dukkakc/Chlamy-EnPhosSite (accessed on 24 April 2023)
DeepPSP	DNN, SENet, Bi-LSTM	?	Uses both local and global sequence information to improve phosphorylation site prediction performance	[79]	https://github.com/DeepPSP (accessed on 24 April 2023)
Predikin 2.0	PSSM	Human	Utilizes the kinase sequence to build scoring matrices based on key residues in the kinase catalytic domain that are known from structural analysis to interact with the substrate phosphorylation site.	[80]	http://predikin.biosci.uq.edu.au (accessed on 24 April 2023)
KinasePhos2.0	SVM	Human?	Predicts phosphorylation sites based on protein sequence profile and protein coupling pattern and the type of kinase that acts at each site	[81]	http://kinasephos2.mbc.nctu.edu.tw/document.html, https://bio.tools/kinasephos_2.0 (accessed on 24 April 2023)
KinasePhos 3.0	SVM, XGBoost	Human and others	Provides comprehensive annotations of kinase-specific phosphorylation sites on multiple proteins. Shapley additive explanations (SHAP) was integrated to increase the feature interpretability	[82]	https://awi.cuhk.edu.cn/KinasePhos/index.html, https://github.com/tom-209/KinasePhos-3.0-executable-file (accessed on 24 April 2023)
DISPHOS (disorder-enhanced phosphorylation predictor)	BLR	Human	Position-specific amino acid frequencies and disorder information is used to improve the discrimination between phosphorylation and non-phosphorylation sites	[83]	http://www.ist.temple.edu/DISPHOS (accessed on 24 April 2023)
pkaPS	Scoring function	Human	Phosphorylation sites of PKA	[84]	http://mendel.imp.univie.ac.at/sat/pkaPS (accessed on 24 April 2023)
Quokka	Seqeunce scoring function + LR	Human	Predicts kinase-specific phosphorylation sites	[85]	http://quokka.erc.monash.edu/ (accessed on 24 April 2023)
PHOSIDA (phosphorylation site database)	SVM	Multiple	Structural and evolutionary investigation and prediction of phosphosites	[45]	http://phosida.de/ (accessed on 24 April 2023)
VPTMdb	SVM, NB, RF	Virus	Predicts viral p-Ser	[47]	http://vptmdb.com:8787/VPTMdb/ (accessed on 24 April 2023)

## Data Availability

Not applicable.
